# PSMD14 as a translational target in cancer and beyond: from deubiquitination mechanisms to drug resistance and precision therapy

**DOI:** 10.20517/cdr.2026.80

**Published:** 2026-09-07

**Authors:** Hongrui Liu, Liang Ma, Yixuan Wang, Yuheng Wang, Siyu Chen, Mingzhen Wang, Dingge Cao, Yi Chen, Wenzhe Si

**Affiliations:** ^1^Department of Laboratory Medicine, Key Laboratory of Cardiovascular Molecular Biology and Regulatory Peptides, State Key Laboratory of Vascular Homeostasis and Remodeling, Peking University Third Hospital, Beijing 100191, China.; ^2^Peking University Fifth School of Clinical Medicine, Beijing 100730, China.; ^3^Tsinghua Shenzhen International Graduate School (SIGS), Tsinghua University, Shenzhen 518055, Guangdong, China.; ^4^Department of Radiation Oncology, Peking University Third Hospital, Beijing 100191, China.; ^#^These authors contributed equally to this work.

**Keywords:** Deubiquitinase, PSMD14, drug resistance, translational medicine, precision therapy, biomarkers

## Abstract

The deubiquitinase PSMD14 (also known as RPN11 or POH1), a critical component of the JAB1/MPN/Mov34 metalloenzyme family, has emerged as a pivotal regulator of protein homeostasis through its deubiquitinating activity. While preclinical studies have extensively characterized PSMD14-mediated stabilization of oncogenic substrates involved in cell cycle progression, programmed cell death, metastasis, metabolic reprogramming, and immune evasion, a translational gap remains between these mechanistic insights and clinical application. This review synthesizes current evidence demonstrating that PSMD14 drives therapeutic resistance across multiple malignancies, including resistance to cisplatin, oxaliplatin, temozolomide, anlotinib, tamoxifen, and bortezomib by deubiquitinating and stabilizing key effectors such as E2F1, ALK2, IMPDH2, estrogen receptor α, and proteasomal components. Furthermore, we highlight the prognostic value of PSMD14 as a biomarker for overall survival and recurrence prediction in hepatocellular carcinoma, pancreatic cancer, lung cancer, and other malignancies, along with its emerging role in non-tumor conditions such as glucocorticoid-induced osteoporosis and post-cardiac arrest neurological outcomes. We critically evaluate the therapeutic landscape of PSMD14 inhibitors, from natural products (thiolutin) and synthetic agents (Capzimin, O-phenanthroline) to next-generation dual-target inhibitors, and discuss the clinical barriers to translation, including off-target toxicity, patient stratification, and optimal combination strategies. By integrating mechanistic discovery with biomarker development and inhibitor optimization, this review aims to lay a foundation for the future development of PSMD14-targeted therapeutic strategies.

## INTRODUCTION

Ubiquitin is a 76-amino-acid protein of approximately 8.5 kDa that plays critical roles in various biological processes in cells^[1]^. Ubiquitination is an important and widespread post-translational modification (PTM) in eukaryotes^[2]^, whereby ubiquitin molecules are conjugated to target proteins through the coordinated action of multiple enzymes, thereby modulating target protein function, localization, stability, and intermolecular interactions^[3]^. The ubiquitination process is typically executed sequentially by three main enzyme classes: ubiquitin-activating enzymes (E1), ubiquitin-conjugating enzymes (E2), and ubiquitin ligases (E3). First, the E1 enzyme uses adenosine triphosphate (ATP) to activate ubiquitin, forming a high-energy thioester bond and transferring it to the active site of an E2 enzyme. Subsequently, the E3 enzyme serves as a substrate-recognition factor, binding the target protein and facilitating the transfer of ubiquitin from the E2 enzyme to a lysine residue on the substrate, thereby forming an isopeptide bond^[4]^.

Ubiquitination is a dynamic and reversible process. Deubiquitinating enzymes (DUBs) remove ubiquitin or polyubiquitin chains from target proteins by hydrolyzing the isopeptide bonds between ubiquitin and substrate protein, thereby reversing the ubiquitination process^[5]^. DUBs comprise multiple enzyme families, including the ubiquitin-specific protease family (USPs), the ovarian tumor protease family (OTUs), the JAB1/MPN/Mov34 metalloenzyme (JAMM) family, the ubiquitin C-terminal hydrolase family (UCHs), and the Machado-Joseph disease protein domain protease family (MJDs), among others^[6-8]^.

The DUB PSMD14 (26S proteasome non-ATPase regulatory subunit 14), also known as RPN11 or POH1, is a member of the JAMM family. By regulating protein ubiquitination levels, PSMD14 is involved in various physiological and pathological processes, including the cell cycle, apoptosis, autophagy, immune responses, and tumorigenesis. Its functions are largely mediated through the specific deubiquitination of diverse substrate proteins, thereby modulating their stability^[9]^. In recent years, accumulating evidence has demonstrated that aberrant expression of PSMD14 is closely associated with multiple human diseases. In the context of malignant tumors, PSMD14 is upregulated in various cancers, including hepatocellular carcinoma (HCC)^[10]^, osteosarcoma^[11]^, head and neck squamous cell carcinoma^[12]^, breast cancer^[13]^, and lung adenocarcinoma^[14]^, where its high expression correlates with poor prognosis. Furthermore, PSMD14 plays important roles in non-tumor diseases. For example, in glucocorticoid-induced osteoporosis, PSMD14 stabilizes SLC7A11 to suppress osteocyte ferroptosis and ameliorate bone loss^[15]^. In the prognostic assessment of brain injury following cardiac arrest, PSMD14 has been validated as a predictive biomarker for early neurological outcomes^[16]^ and in choroidal neovascularization, PSMD14 regulates the Keap1/Nrf2 antioxidant pathway by deubiquitinating and stabilizing Keap1^[17]^. These findings suggest that the functions of PSMD14 are not limited to tumor regulation but extend broadly to the pathological processes of various diseases.

This review focuses on PSMD14-driven mechanisms of therapeutic resistance in cancer, while also considering its broader pathophysiological roles in certain non-malignant conditions that may contribute to target validation and safety assessment, particularly with respect to its deubiquitinating substrates and functions. Furthermore, we discuss the clinical value of PSMD14 as a prognostic biomarker and recent advances in therapeutic strategies targeting PSMD14, aiming to provide new insights for the development of precision therapeutic approaches.

## BIOLOGICAL PROCESSES AFFECTED BY PSMD14

**Figure 1:**
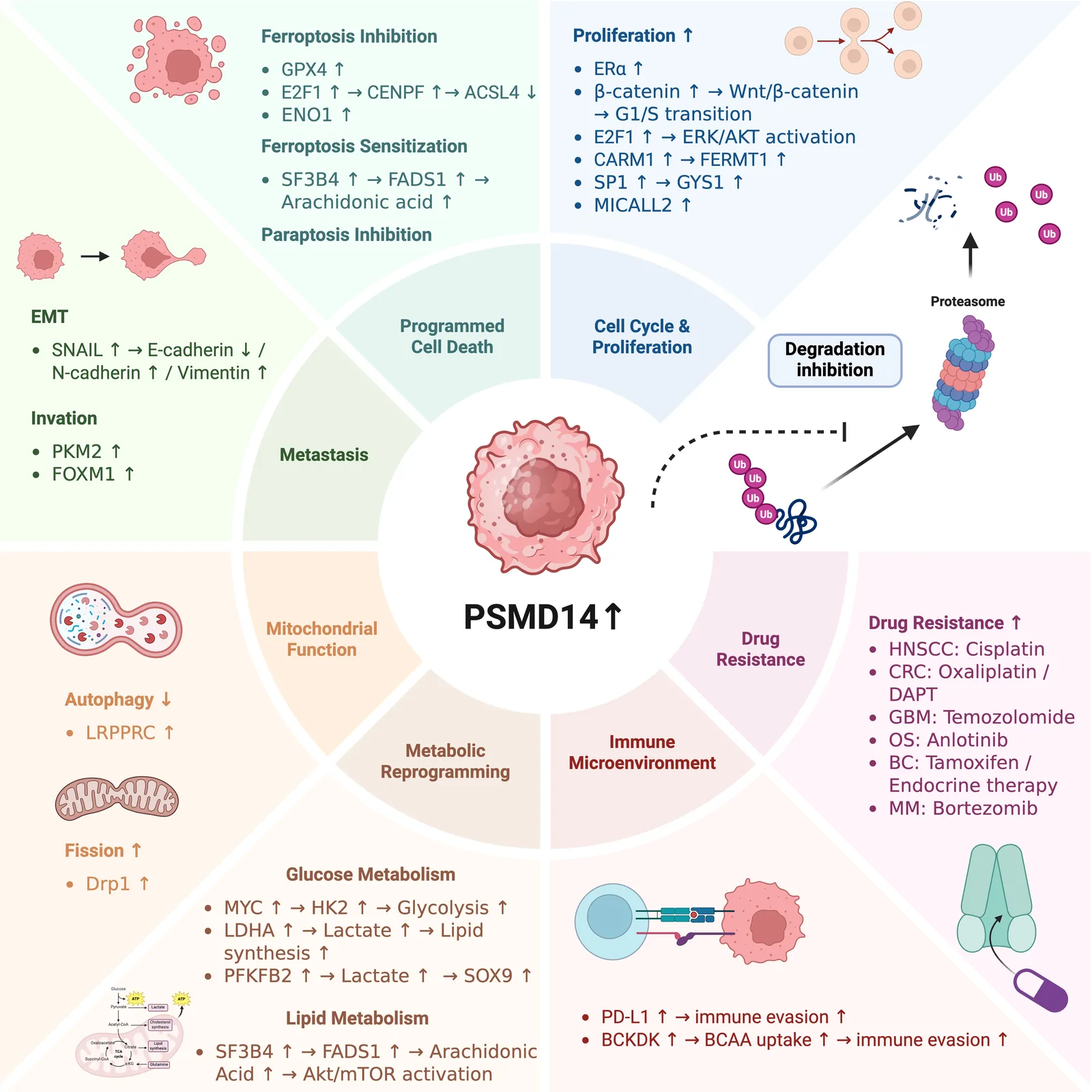
Overview of PSMD14-mediated mechanisms driving tumor progression and therapeutic resistance. PSMD14 deubiquitinates and stabilizes a broad range of substrate proteins, thereby regulating cell cycle progression, programmed cell death (ferroptosis and paraptosis), EMT, invasion and metastasis, mitochondrial function, and metabolic reprogramming. These concerted actions ultimately drive therapeutic resistance to chemotherapy, targeted or endocrine therapy, and proteasome inhibitors across multiple cancer types. Arrows indicate regulatory relationships, and ↑/↓ denote activation or suppression of the corresponding pathways or phenotypes. Created in BioRender. Liu, H. (2026) https://BioRender.com/j66f63i. EMT: Epithelial-mesenchymal transition; ERα: estrogen receptor α; AKT: protein kinase B; MYC: MYC proto-oncogene; HK2: hexokinase 2; LDHA: lactate dehydrogenase A; mTOR: mechanistic target of rapamycin; BCAA: branched-chain amino acid; HNSCC: head and neck squamous cell carcinoma; CRC: colorectal cancer; GBM: glioblastoma; OS: osteosarcoma; BC: breast cancer; MM: multiple myeloma.

PSMD14 deubiquitinates and stabilizes a broad range of substrate proteins, thereby regulating multiple biological processes as summarized in Figure 1.

### Regulation of the cell cycle and proliferation

PSMD14 plays a central role in regulating the cell cycle and driving tumor cell proliferation by directly deubiquitinating and stabilizing a diverse set of pro-proliferative proteins. In estrogen receptor (ER)-positive breast cancer cell lines (MCF-7, T47D), PSMD14 inhibits K48-linked ubiquitination of estrogen receptor α (ERα), which would otherwise target ERα for proteasomal degradation. By stabilizing ERα protein levels, PSMD14 promotes cell cycle progression and thus enhances tumor cell proliferation^[18]^. Similarly, in glioblastoma cell lines (U251, T98G), PSMD14 promotes tumorigenesis by stabilizing β-catenin, activating the Wnt/β-catenin signaling pathway, upregulating cyclin D1 expression, thereby facilitating the G1/S transition, and driving glioblastoma cell proliferation^[19]^. In another example, PSMD14 increases the stability of E2F1 by directly binding to and deubiquitinating this transcription factor in anaplastic thyroid carcinoma (ATC) cell lines (8505C, CAL62), thereby promoting tumor cell proliferation^[20]^. Furthermore, PSMD14 can stabilize target enzymes as demonstrated in hepatocellular carcinoma cell lines (Huh7, PLC/PRF/5, SK-Hep-1, MHCC-97H), where PSMD14 mediates the deubiquitination of CARM1, induces transcriptional activation of FERMT1, and consequently promotes hepatocellular carcinoma cell proliferation^[21]^. In melanoma cell lines (A375, SK-MEL-28), PSMD14 promotes proliferation through the PSMD14-SP1-GYS1 axis: it directly interacts with and deubiquitinates SP1 by removing K48-linked polyubiquitin chains, thereby enhancing its protein stability; stabilized SP1 subsequently transactivates GYS1, a key enzyme in glycogen synthesis, to drive melanoma cell proliferation and migration^[22]^. Notably, PSMD14 itself can also be regulated by PTMs. In clear cell renal cell carcinoma cell lines (KMRC-1, OS-RC-2), PSMD14 is upregulated and promotes malignant progression through a phosphorylation-dependent mechanism. The dual-specificity phosphatase DUSP4 dephosphorylates PSMD14 at Y32, which enhances the binding affinity between PSMD14 and MICALL2. Consequently, PSMD14 removes ubiquitin chains from MICALL2 and prevents its degradation, thereby promoting clear cell renal cell carcinoma (ccRCC) cell proliferation, migration, and invasion^[23]^.

### Regulation of programmed cell death (ferroptosis and paraptosis)

PSMD14 plays a critical role in regulating programmed cell death, largely by suppressing both ferroptosis and paraptosis to promote tumor cell survival.

In the context of ferroptosis, PSMD14 inhibits this process through multiple mechanisms. In bladder cancer cell lines (T24, 5637), PSMD14 depletion suppresses cell proliferation by downregulating GPX4, which is associated with enhanced ferroptosis^[24]^. In triple-negative breast cancer, PSMD14 exerts dual regulation of arachidonic acid (AA) metabolism to modulate ferroptosis. On one hand, in triple-negative breast cancer cell lines (Hs578T, MDA-MB-231), PSMD14 deubiquitinates and stabilizes E2F1, which then transcriptionally upregulates CENPF; CENPF in turn inhibits ACSL4 (a key positive regulator of ferroptosis), reduces AA metabolite accumulation, and thereby attenuates ferroptotic cell death^[25]^. In triple-negative breast cancer cell lines (MDA-MB-231, BT-549), PSMD14 deubiquitinates and stabilizes SF3B4, promoting HNRNPC/SF3B4 complex-mediated splicing of FADS1 mRNA and upregulating FADS1 expression. FADS1 is a key enzyme in AA biosynthesis; its upregulation increases intracellular AA content, thereby sensitizing cancer cells to ferroptosis^[26]^. Together, these two studies reveal how PSMD14 finely tunes AA metabolism to modulate ferroptosis from distinct yet complementary angles. In the intrahepatic cholangiocarcinoma cell line (HuCCT1), an additional layer of ferroptosis regulation has been uncovered: L-lactate, produced by enhanced glycolysis, drives PSMD14 lactylation at lysine 100 (K100), which delays proteasome-mediated degradation and increases PSMD14 stability. Elevated PSMD14 subsequently interacts with ENO1 and removes K63-linked ubiquitin chains, thereby inhibiting lysosome-mediated ENO1 degradation and suppressing ferroptosis. Importantly, pharmacological inhibition of PSMD14 with Capzimin sensitizes intrahepatic cholangiocarcinoma (ICC) cells to ferroptosis and synergizes with anti-programmed cell death protein 1 (anti-PD-1) therapy *in vivo*, highlighting the therapeutic potential of targeting this axis^[27]^. Furthermore, the role of PSMD14 in suppressing ferroptosis extends beyond cancer: in an osteocyte-like cell line (MLO-Y4), PSMD14 stabilizes SLC7A11, thereby suppressing osteocyte ferroptosis and ameliorating glucocorticoid-induced osteoporosis^[15]^.

In the context of paraptosis, PSMD14 similarly inhibits cell death. Paraptosis is a non-apoptotic form of programmed cell death characterized by extensive vacuolization of the endoplasmic reticulum and mitochondria. In breast cancer cell lines (MDA-MB-435S, BT-549, MDA-MB-468), pharmacological inhibition of PSMD14 leads to proteasome inhibition, endoplasmic reticulum stress, and calcium imbalance through multiple mechanisms, ultimately triggering paraptosis in breast cancer cells^[28]^. This suggests that under physiological conditions, PSMD14 may maintain proteasome function to prevent paraptosis.

### Regulation of invasion- and metastasis-related genes

PSMD14 influences tumor cell migration and metastasis by regulating epithelial-mesenchymal transition (EMT)-related genes, as well as other invasion- and metastasis-associated factors. EMT is a biological process through which epithelial cells acquire a mesenchymal phenotype, thereby gaining enhanced migratory and invasive capabilities.

In the context of EMT regulation, PSMD14 promotes EMT by stabilizing multiple substrate proteins. In anaplastic thyroid carcinoma, PSMD14 knockdown upregulates E-cadherin, downregulates N-cadherin and vimentin, reverses the EMT phenotype, and consequently inhibits metastasis^[20]^. In esophageal squamous cell carcinoma (ESCC) cell lines (KYSE30, KYSE510), PSMD14 directly binds to and deubiquitinates SNAIL, thereby stabilizing it; accordingly, PSMD14 knockdown upregulates E-cadherin, downregulates N-cadherin and vimentin, blocks SNAIL-induced EMT, and suppresses tumor metastasis^[29]^. In lung cancer cell lines (H1299, A549), PSMD14 stabilizes SMAD3 through deubiquitination, which in turn activates the TGF-β signaling pathway and promotes EMT and tumor metastasis^[30]^.

Beyond EMT-related proteins, PSMD14 also modulates tumor cell invasion and metastasis through non-EMT pathways by interacting with other substrates. For example, in ovarian cancer cell lines (A2780, OVCAR-3, and HO-8910), PSMD14 deubiquitinates PKM2 to promote aerobic glycolysis, thereby conferring metabolic plasticity for tumor cell migration and invasion^[31]^. In breast cancer cell lines (MDA-MB-468, MDA-MB-231, BT-549, T-47D), PSMD14 promotes malignant progression through two distinct mechanisms: deubiquitinating and stabilizing FOXM1 and activating the phosphoinositide 3-kinase (PI3K)/protein kinase B (AKT)/mechanistic target of rapamycin (mTOR) pathway^[32]^.

### Regulation of mitochondrial function

PSMD14 exerts pro-tumorigenic effects in multiple cancers by deubiquitinating and stabilizing mitochondria-associated proteins, thereby regulating mitochondrial fission, dynamics, and mitophagy. In ovarian cancer cell lines (Caov3, A2780, SKOV3, OVCAR3), PSMD14 deubiquitinates and stabilizes LRPPRC, thereby inhibiting autophagy and promoting ovarian cancer progression^[33]^. Additionally, in ovarian cancer cell lines (SKOV3, A2780), HAPSTR1 recruits PSMD14 to interact with LRPPRC, further enhancing LRPPRC stability and promoting ovarian cancer progression^[34]^. In contrast, in bladder cancer cell lines (T24, 5637), PSMD14 deubiquitinates and stabilizes Drp1, thereby preventing its proteasomal degradation, enhancing Drp1 activity, and promoting mitochondrial fission, which consequently accelerates bladder cancer cell proliferation^[35]^. Together, these findings demonstrate that PSMD14 can promote tumor progression through distinct mitochondrial regulatory mechanisms - either by inhibiting autophagy or promoting mitochondrial fission - depending on the cellular context and substrate availability.

### Metabolic reprogramming

PSMD14 contributes to metabolic reprogramming in tumor cells by modulating the stability of key metabolic enzymes. In glucose metabolism, PSMD14 enhances aerobic glycolysis and thereby promotes tumor progression. In pancreatic cancer cell lines (PANC1, AsPC1), POH1 (PSMD14) facilitates the initiation and progression of pancreatic ductal adenocarcinoma (PDAC) by deubiquitinating and stabilizing MYC, which upregulates hexokinase 2 (HK2) expression, promotes glycolysis, and drives metabolic reprogramming^[36]^. Beyond transcriptional regulation of glycolytic enzymes, PSMD14 also orchestrates a deubiquitination-metabolism-epigenetic cascade in pancreatic cancer cell lines (PANC-1, MIA PaCa-2): it directly deubiquitinates and stabilizes lactate dehydrogenase A (LDHA), leading to lactate accumulation and subsequent H3K18 lactylation, which epigenetically activates adenosine triphosphate (ATP) citrate lyase (ACLY) transcription and drives lipid synthesis to promote tumor progression^[37]^. In gastric adenocarcinoma cell lines (AGS, BGC823, HGC-27), PSMD14 deubiquitinates PFKFB2 at K355, which subsequently promotes SCYL2-mediated phosphorylation of PFKFB2, leading to enhanced glycolysis and lactate accumulation. Accumulated lactate then activates H3K27 lactylation, which in turn upregulates PSMD14 and SOX9 expression, forming a positive feedback loop that drives cancer stemness^[38]^.

In lipid metabolism, PSMD14 deubiquitinates and stabilizes SF3B4 in triple-negative breast cancer cell lines (MDA-MB-231, BT-549), promoting alternative splicing and expression of FADS1 mRNA, driving AA synthesis, activating the Akt/mTOR signaling pathway, remodeling the lipid metabolic network, and thereby facilitating metabolic reprogramming and tumor progression in triple-negative breast cancer^[26]^.

### Regulation of the immune microenvironment

PSMD14 has been implicated as a key regulator of the tumor immune microenvironment, modulating immune cell infiltration, immune checkpoint molecule expression, and cytokine signaling pathways, thereby promoting an immunosuppressive microenvironment that facilitates immune evasion and disease progression.

Clinical and bioinformatics studies have established correlations between PSMD14 expression and immune cell infiltration patterns in multiple cancer types. In hepatocellular carcinoma, PSMD14 serves as a core component of an immune-related gene prognostic index (IRGPI). This index is significantly correlated with the infiltration levels of multiple immune cell populations, including B cells, CD4^+^ T cells, CD8^+^ T cells, dendritic cells, neutrophils, and macrophages, and can predict poor prognosis^[39]^. In lung adenocarcinoma, bioinformatics analysis has revealed that PSMD14 expression is negatively correlated with the expression of immune checkpoint molecules such as PD-1 and TIGIT, is associated with increased infiltration of immunosuppressive Th2 cells, and is correlated with reduced function of effector natural killer (NK) cells, suggesting an association with an immunosuppressive microenvironment that facilitates immune escape and contributes to poor prognosis^[40]^.

Beyond correlative associations, mechanistic studies have revealed that PSMD14 directly regulates immune checkpoint molecules through PTMs. In breast cancer cell lines (MDA-MB-231, MDA-MB-468, BT-549), PSMD14 interacts with the intracellular domain of programmed death-ligand 1 (PD-L1) and removes K48-linked polyubiquitin chains, thereby inhibiting proteasomal degradation and stabilizing PD-L1 expression. PSMD14 knockdown reduces PD-L1 levels and enhances CD8^+^ T-cell-mediated cytotoxicity, as evidenced by increased granzyme B and IFN-γ release. Furthermore, PSMD14 inhibition reshapes the tumor microenvironment by reducing regulatory T cell and myeloid-derived suppressor cell accumulation while promoting M1 macrophage polarization. *In vivo*, PSMD14 knockdown synergizes with anti-PD-1 therapy to suppress tumor growth in a 4T1 syngeneic mouse model^[41]^.

In addition, PSMD14 can indirectly modulate immune responses by regulating metabolic pathways and signaling axes within the tumor microenvironment. In glioblastoma cell lines (A172, U251), PSMD14 has been identified as a key regulator of metabolic immune evasion: it stabilizes BCKDK through deubiquitination, promoting branched-chain amino acid (BCAA) uptake by tumor cells and leading to BCAA depletion in the tumor microenvironment, which in turn suppresses NK and CD8^+^ T cell cytotoxicity; pharmacological inhibition of PSMD14 with O-phenanthroline (OPA) restores immune cell infiltration and enhances chimeric antigen receptor (CAR)-NK cell therapy efficacy^[42]^.

### Regulation of tumor drug resistance

PSMD14 plays a critical role in the development of resistance to anticancer therapies through multiple mechanisms, promoting resistance to chemotherapy, targeted/endocrine therapy, and proteasome inhibitors.

In the context of chemotherapy resistance, PSMD14 mediates resistance to chemotherapeutic agents via distinct substrates. In head and neck squamous cell carcinoma cell lines (SCC15, UM1), PSMD14 stabilizes E2F1 through deubiquitination, activates the Akt/SOX2 signaling axis, and maintains cancer stem cell properties, thereby promoting resistance to cisplatin; conversely, PSMD14 inhibition antagonizes this signaling axis and overcomes cisplatin resistance^[43]^. In colorectal cancer cell lines (HCT116, RKO), PSMD14 stabilizes the ALK2 receptor, activates the BMP6 signaling pathway, and upregulates ATP-binding cassette (ABC) transporter expression (ABCA7/ABCC4), thereby enhancing resistance to oxaliplatin and DAPT, while preserving cancer stem cell properties^[44]^. In glioblastoma cell lines (LN229, A172, U118MG) and patient-derived GBM stem-like cells (GBM#P3), PSMD14 stabilizes IMPDH2 (the rate-limiting enzyme of *de novo* purine biosynthesis) by removing K48-linked polyubiquitin chains, thereby maintaining nucleotide metabolism and mitochondrial function, and promotes resistance to temozolomide; furthermore, treatment with the PSMD14 inhibitor thiolutin (THL) synergizes with temozolomide to suppress tumor growth^[45]^.

In the context of targeted and endocrine therapy resistance, PSMD14 also plays a critical role. In osteosarcoma cell lines (U2OS, Saos-2), PSMD14 activates the PI3K/Akt/mTOR signaling pathway and promotes resistance to the multi-target tyrosine kinase inhibitor anlotinib; knockdown of PSMD14 inhibits proliferation, migration, and invasion of resistant cell lines and reverses their resistance^[46]^. In ER-positive breast cancer cell lines (MCF-7, T47D), PSMD14 inhibits K48-linked ubiquitination of ERα, stabilizes ERα expression, and enhances resistance to endocrine therapy; targeted inhibition of PSMD14 promotes degradation of mutant ERα (such as Y537S) and restores sensitivity to tamoxifen and other endocrine agents^[18]^.

**Figure 2:**
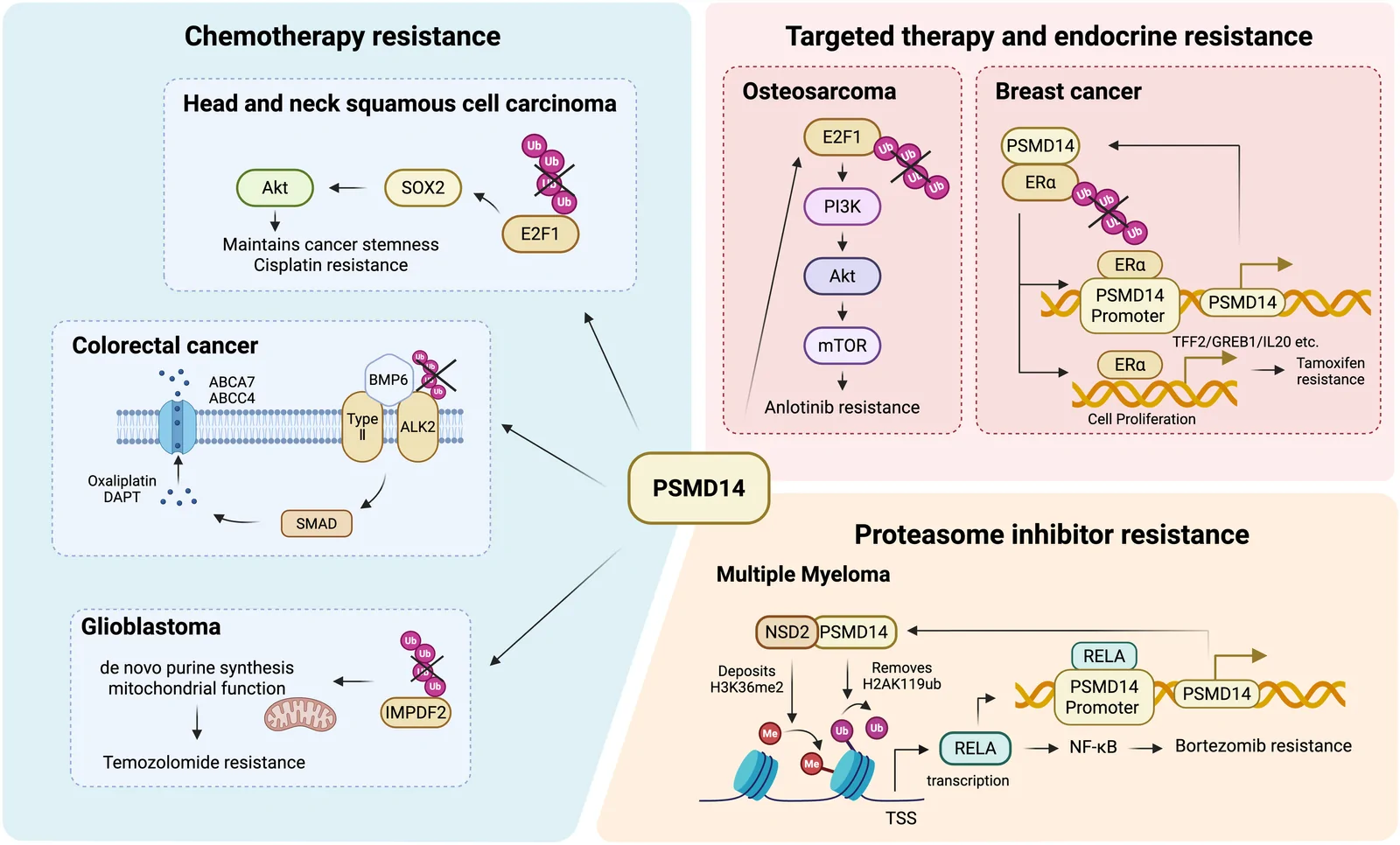
Major mechanisms of PSMD14-mediated tumor drug resistance. Based on its substrate specificity, PSMD14 promotes resistance to various therapeutic modalities through distinct signaling pathways. In the context of chemotherapy resistance, PSMD14 promotes cisplatin resistance by stabilizing E2F1 in head and neck squamous cell carcinoma, which maintains cancer stemness; enhances resistance to oxaliplatin and DAPT by stabilizing the ALK2 receptor in colorectal cancer, which activates the BMP6 signaling pathway and upregulates ABC transporters; and drives temozolomide resistance by stabilizing IMPDH2 in glioblastoma, which sustains de novo purine synthesis and mitochondrial function. In the context of targeted and endocrine therapy resistance, PSMD14 promotes anlotinib resistance by activating the PI3K/Akt/mTOR pathway in osteosarcoma, and enhances tamoxifen resistance by stabilizing ERα in breast cancer, which potentiates estrogen signaling transcription. In the context of proteasome inhibitor resistance, PSMD14 drives bortezomib resistance in multiple myeloma by functioning as a histone deubiquitinase (H2AK119ub) and cooperating with NSD2 to activate RELA/NF-κB transcription. Created in BioRender. Liu, H. (2026) https://BioRender.com/k88t3mg. ABC: ATP-binding cassette; PI3K: phosphoinositide 3-kinase; Akt: protein kinase B; mTOR: mechanistic target of rapamycin; ERα: estrogen receptor α; NSD2: nuclear receptor binding SET domain protein 2; RELA: RELA proto-oncogene, NF-κB subunit; NF-κB: nuclear factor kappa-light-chain-enhancer of activated B cells.

In the context of proteasome inhibitor resistance, PSMD14 also plays an important role. As early as 2017, studies in multiple myeloma cell lines, including bortezomib-sensitive MM.1S and bortezomib-resistant ANBL6.BR demonstrated that pharmacological inhibition of PSMD14 with OPA blocks proteasome function, induces apoptosis, and overcomes bortezomib resistance; OPA exhibits synergistic anti-tumor activity when combined with lenalidomide, pomalidomide, or dexamethasone^[47]^. Subsequent studies revealed that in multiple myeloma cell lines (LP-1, KMS11), PSMD14 functions as a histone deubiquitinase (H2AK119ub) on chromatin, cooperating with the histone methyltransferase NSD2 to activate transcription of oncogenes such as RELA; RELA in turn transactivates PSMD14, forming a positive feedback loop that drives myelomagenesis and drug resistance. PSMD14 inhibition enhances sensitivity to bortezomib^[48]^. The major mechanisms of PSMD14-mediated drug resistance across different cancer types are summarized in Figure 2.

## VALUE OF PSMD14 AS A PROGNOSTIC BIOMARKER

In recent years, the oncogenic functions of the deubiquitinase PSMD14 have been documented in a wide range of cancer types. A systematic review and meta-analysis by Dong *et al.*, encompassing 8 studies and 1,608 patients, demonstrated that high PSMD14 expression is significantly associated with reduced overall survival [OS; pooled hazard ratio (HR) = 1.92, 95% confidence interval (CI) 1.67-2.22, *P* < 0.00001] and disease-free survival (pooled HR = 1.84, 95%CI 1.33-2.55, *P* = 0.0003). Elevated PSMD14 expression was also correlated with larger tumor size, poorer differentiation, and a higher incidence of lymph node metastasis^[49]^. Within this pan-cancer context, tumor type–specific studies have further elucidated the prognostic relevance of PSMD14.

To date, multiple studies have assessed the relationship between PSMD14 expression and patient outcomes using Kaplan–Meier survival analyses. In PDAC^[50]^ (*P* < 0.05), osteosarcoma^[11]^ (*P* = 0.013), non-small cell lung cancer^[51]^ (*P* < 0.001), lung adenocarcinoma^[40]^ (*P* = 0.026), and head and neck squamous cell carcinoma^[12]^ (*P* = 0.0132), high PSMD14 expression correlates with shorter OS, indicating an unfavorable prognosis.

Beyond oncology, PSMD14 has also been investigated as a predictor of early postoperative neurological outcomes in patients with cardiac arrest. In a prognostic model for brain injury following cardiac arrest, PSMD14 was identified as the most readily detectable peripheral blood biomarker candidate, with the highest area under the curve (AUC, 0.767). Its protein level showed a strong positive linear correlation with NDS neurological function scores (r ≈ 0.89, *P* < 0.001), and PSMD14 was validated for the first time as a predictor of early neurological outcome in patients who underwent out-of-hospital resuscitation^[16]^. In addition, a bioinformatics study on non-syndromic Tetralogy of Fallot, a congenital heart disease, identified PSMD14 as a potential hub gene involved in proteostasis dysregulation during right ventricular outflow tract remodeling, further suggesting its broader pathophysiological relevance beyond cancer^[52]^.

Multivariate Cox regression analyses have further indicated that PSMD14 serves as an independent prognostic factor for OS in pancreatic cancer^[50]^ (HR = 3.849, 95%CI 2.118-6.995, *P* = 0.001) and non-small cell lung cancer^[51]^ (HR = 1.679, 95%CI 1.086-2.596, *P* = 0.020). In addition, multiple studies have constructed multigene prognostic risk score models incorporating PSMD14 expression using multivariate Cox regression analyses. The predictive performance of these models varied considerably across cancer types, as summarized in Table 1^[39,53-58]^. Notably, the glioma model achieved relatively high AUC values (0.818-0.861), whereas models for hepatocellular carcinoma and cervical cancer showed moderate predictive performance, with AUC values of 0.694-0.901 and 0.676-0.730, respectively. In contrast, the breast cancer risk model exhibited more limited predictive performance, with 1- to 3-year AUC values ranging from 0.61 to 0.64, indicating that the predictive value of PSMD14-containing multigene signatures varies across cancer types and would require further optimization before broad clinical application.

**Table 1:** Multigene predictive and risk models incorporating PSMD14 expression across different diseases and conditions

**Disease type**	**Components of prognostic risk score**	**Gene category**	Samples*	**Method**	**AUC**	**Ref.**
Hepatocellular carcinoma	HSPA4 × 0.022 + **PSMD14 × 0.042** + RBP2 × 0.019 + MAPT × 0.197 + TRAF3 × 0.146 + NDRG1 × 0.006 + NRAS × 0.027 + IL17D × 0.075	Immune-related genes	TCGA: 365 HCC + 50 normal	Multivariate Cox regression analysis	1-/3-/5-year: 0.721/0.747/0.781	[53]
Hepatocellular carcinoma	**PSMD14 × 0.0813** + FABP6 × 0.1172 + ISG20L2 × 0.1264 + HGF × 0.1264 + BIRC5 × 0.0628 + IL17D × 0.0847 + STC2 × 0.0382	Immune-related genes	TCGA: 374 HCC + 50 normal	Univariate and multivariate Cox regression analyses	Training/test: 0.892/0.727; ICGC validation: 0.694	[39]
Hepatocellular carcinoma (Asian population)	MICB × 0.204138821 + **PSMD14 × 0.132083415** + TRAF3 × 0.347452557 + SP1 × (-0.186333147) + NDRG1 × 0.019496354 + HDAC1 × (-0.038473968) + HRAS × 0.038113834 + NRAS × 0.088094449 + SEMA5B × 0.649885818 + GMFB × 0.12019319 + ACVR2B × 0.528385329 + BRD8 × 0.176246154 + MMP12 × (-0.213311477) + KITLG × (-0.245472873) + DCK × 0.141294643	Immune-related genes	TCGA: 160 tumor + 6 normal	Univariate and multivariate Cox regression prognostic analyses	0.901	[54]
Glioma	PSMB6 × (-0.5071) + PSMD9 × 0.3068 + UBB × 0.3587 + PSMD12 × 0.9338 + PSMB10 × 0.2287 + PSMA5 × 0.7667 + **PSMD14 × 0.0892**	Hypoxia-related genes	TCGA: 663 glioma; GTEx: 2,642 normal	LASSO model construction; univariate and multivariate Cox regression analyses	1-/3-/5-year: 0.818/0.861/0.830	[55]
Cervical cancer	**PSMD14 × 0.54** + PSMA4 × (-0.74) + ZBTB16 × (-0.42) + RADD × (-0.021) + ANKRD9 × 0.54	E3 ubiquitin ligase–associated genes	TCGA-CESC: 306 tumor + 3 normal; GEO: 300 tumor	LASSO and Cox regression analyses	TCGA (1-/3-/5-year): 0.676/0.700/0.698; GSE44001 (1-/3-/5-year): 0.694/0.730/0.702	[56]
Breast cancer	PSME2 × (-0.36229220) + PSMB8 × (-0.06306154) + **PSMD14 × 0.43677028**	Polyamine metabolism- and immune-related genes	TCGA-BRCA + GSE20685 (GEO): 1,082 tumor + 113 normal	LASSO and Cox regression analyses	TCGA-BRCA (1-/2-/3-year): 0.61/0.61/0.64	[57]
TAO	AGO2 × (-0.17022) + CP × (-0.23335) + DIO3 × 0.11804 + **PSMD14 × (-0.67564)** + WTIP × 0.65642	Hypoxia-related genes	GSE58331 + GSE105149: 12 TAO + 14 control	LASSO and Cox regression analyses	Training: 0.976; Test: 0.692	[58]

*TCGA provides both tumor tissue samples and corresponding expression data; GTEx and GEO provide expression data only. Bold indicates PSMD14 and its coefficient in the risk score formulas. AUC: Area under the curve; TCGA: The Cancer Genome Atlas; HCC: hepatocellular carcinoma; GTEx: Genotype-Tissue Expression; GEO: Gene Expression Omnibus; TAO: thyroid-associated ophthalmopathy.

In addition to OS, the predictive value of PSMD14 for early postoperative recurrence has been systematically evaluated in hepatocellular carcinoma. Xiong *et al.* conducted tissue microarray–based immunohistochemistry in 312 HCC patients who underwent R0 resection and found that high PSMD14 expression in tumor tissues was an independent risk factor for early postoperative recurrence (≤ 24 months) (HR = 2.17, 95%CI 1.42-3.32, *P* = 0.0003). Furthermore, a nomogram integrating clinicopathological parameters showed that inclusion of PSMD14 expression increased the C-index of the model for predicting 1-, 3-, and 5-year recurrence-free survival (RFS) from 0.68 to 0.75 (*P* < 0.01), indicating that PSMD14 markedly improves the accuracy of postoperative recurrence prediction^[59]^.

## PSMD14 INHIBITORS

In recent years, THL has emerged as one of the most extensively studied PSMD14 inhibitors. THL inhibits PSMD14/Rpn11 deubiquitinase activity by chelating Zn^2+^ within the JAMM domain^[60]^. Currently classified as a tool compound at the preclinical stage, this mechanism has been validated in multiple solid tumor models, including breast cancer^[18]^, head and neck squamous cell carcinoma^[43]^, ESCC^[61]^, and anaplastic thyroid cancer^[20]^, in which THL significantly inhibits tumor growth. In ER-positive breast cancer cell lines (MCF-7, T47D), THL inhibits the deubiquitinase activity of PSMD14, directly downregulates ERα protein (by shortening its half-life) and its downstream target genes such as *GREB1*, *TFF1*, and *IL20*, and markedly suppresses cell proliferation^[18]^. In head and neck squamous cell carcinoma cell lines (SCC15, UM1), THL inhibits PSMD14 deubiquitinase activity, accelerates E2F1 degradation, disrupts the E2F1–Akt–SOX2 axis, reduces cancer stemness, and significantly enhances cisplatin sensitivity^[43]^. In ESCC cell lines (KYSE 30, KYSE 150), THL disrupts the interaction between PSMD14 and SNAIL, promotes SNAIL ubiquitination and degradation, upregulates E-cadherin, downregulates N-cadherin and vimentin, and effectively reverses the EMT. Functionally, THL not only suppresses the migration, invasion, and stemness of ESCC cells but also induces apoptosis and synergistically enhances chemosensitivity to cisplatin^[61]^. In anaplastic thyroid carcinoma cell lines (8505C, CAL62), THL similarly targets and inhibits the deubiquitinase activity of PSMD14, promotes E2F1 degradation, and diminishes the transcriptional activation of the PI3K/AKT/mTOR and Ras/Raf/MEK/ERK pathways (as reflected by reduced p-AKT and p-ERK levels). As a result, THL inhibits cell proliferation and migration and induces apoptosis. Concurrently, THL upregulates E-cadherin and downregulates mesenchymal markers such as N-cadherin and vimentin, thereby blocking EMT and reducing the invasive and metastatic capacities of ATC cells^[20]^.

Research on THL has progressed beyond its activity as a natural product and its single-target mechanism, with substantial advances in structural optimization, dual-target design, and improvement in synthetic routes. For ESCC, He *et al.* used the natural product THL as a lead compound, retained its core scaffold responsible for PSMD14 inhibition, and introduced histone deacetylase (HDAC) inhibitory moieties at the N7 position to generate a series of dual-target inhibitors. Among these, compound 8b, which contains an eight-carbon linker and a 1,2-phenylenediamine group, exhibited the most balanced dual-target inhibitory activities (PSMD14 IC_50_ = 238.7 ± 27 nM; HDAC1 IC_50_ = 141.2 ± 10.3 nM). In the ESCC cell line (KYSE 30), dual targeting of PSMD14 and HDAC by 8b destabilizes the oncogenic transcription factor Snail, restores histone H3 acetylation, promotes chromatin condensation and gene silencing, and consequently reverses the EMT phenotype^[62]^. Whether applied as monotherapy or in combination strategies, compound 8b, as the first dual PSMD14/HDAC inhibitor, outperforms single-target PSMD14 or HDAC inhibitors, highlighting the therapeutic advantage of dual-target inhibition. Currently in the early discovery stage, compound 8b requires further pharmacokinetic optimization and comprehensive preclinical evaluation. These findings not only expand the pharmacological potential of THL but also provide a new paradigm for multi-target anticancer drug design. Future work should focus on further structural optimization of THL-based compounds, rational target-combination strategies, and comprehensive preclinical evaluation.

Beyond THL, other PSMD14 inhibitors include 8-thioquinoline (8TQ) and its derivative Capzimin, and 1,10-phenanthroline (OPA). Like THL, these agents function as metal-chelating inhibitors that target the catalytic Zn^2+^ ion within the JAMM domain. As a tool compound, 8TQ exerts potent inhibitory activity against Rpn11 (IC_50_ = 2.8 ± 0.4 μM) by chelating the catalytic Zn^2+^ ion of Rpn11 via its endocyclic nitrogen and exocyclic sulfur donor atoms in a bidentate fashion, thereby blocking deubiquitinating activity and preventing the removal of ubiquitin chains from substrate proteins^[63]^. Through structure–activity relationship (SAR) optimization of 8TQ, Capzimin was developed as a derivative with approximately sevenfold higher potency against Rpn11 (IC_50_ = 0.34 μM). Similar to 8TQ, Capzimin is a noncompetitive inhibitor of the 26S proteasome that directly targets and inhibits the Rpn11•Rpn8 complex. By broadly suppressing protein degradation in diverse cancer cell lines, Capzimin ultimately inhibits cell proliferation and induces apoptosis^[64]^. Currently classified as a validated chemical probe for PSMD14/Rpn11 with moderate cell-based activity (B-level probe), Capzimin has demonstrated anti-tumor efficacy in preclinical models of multiple malignancies. Notably, in lung adenocarcinoma cell lines (H1299, PC9, A549, H1975), Capzimin exhibited potent anti-tumor effects with IC_50_ values ranging from 3.4 to 8.3 μM, suppressing proliferation, migration, and invasion while inducing mitochondrial-mediated apoptosis. *In vivo*, Capzimin significantly suppressed tumor growth in xenograft mouse models, and its combination with the TGF-β inhibitor galunisertib demonstrated enhanced efficacy^[65]^. In prostate cancer cell lines (PC3, DU145, 22RV1, and LNCaP), Capzimin targets POH1, suppresses proliferation, induces cell cycle arrest, and significantly enhances the efficacy of chemotherapy and androgen deprivation therapy, suggesting its potential as a novel targeted therapeutic, particularly for refractory prostate cancer^[66]^.

OPA specifically binds to and inhibits the metalloprotease activity of Rpn11 without affecting other deubiquitinases. In multiple myeloma, high PSMD14 expression is strongly associated with poor prognosis and resistance to the proteasome inhibitor bortezomib. Currently classified as a tool compound at the preclinical stage, OPA targets the deubiquitinase activity of the 19S proteasome, blocks proteasome function, activates caspase-dependent apoptosis and endoplasmic reticulum stress signaling, effectively overcoming bortezomib resistance, and potentiates the synergistic antitumor effects of immunomodulatory drugs (such as lenalidomide and pomalidomide) and dexamethasone^[47]^. Beyond hematological malignancies, OPA has also demonstrated efficacy in solid tumors: in glioblastoma, OPA treatment restores immune cell infiltration and enhances CAR-NK cell cytotoxicity by disrupting the PSMD14-BCKDK-BCAA metabolic axis^[42]^. Recent advances in nanomedicine have expanded the therapeutic potential of OPA. An acid-responsive cobalt-doped ZIF-8 nanoplatform encapsulating OPA (CPP) has been developed to enable tumor-selective delivery and pH-triggered release. Upon exposure to the acidic tumor microenvironment, CPP releases both Co^2+^, which catalyzes Fenton-like reactions to generate reactive oxygen species (ROS), and OPA, which inhibits PSMD14 and promotes SLC7A11 ubiquitination and degradation, thereby depleting glutathione and sensitizing tumor cells to ferroptosis. In osteosarcoma xenograft models, CPP administration significantly suppressed tumor growth, demonstrating the therapeutic potential of combining PSMD14 inhibition with nanocarrier-based drug delivery^[67]^.

In addition to these metal-chelating inhibitors, researchers have also explored covalent inhibitors that irreversibly target PSMD14. Epidithiodiketopiperazines (ETPs) inhibit PSMD14 and thereby compromise proteasome function. However, ETPs can also inhibit other JAMM family proteins, including CSN5 and AMSH. Currently in the early discovery stage, SOP11 is a modified ETP with reduced nonspecific effects that stabilizes a subset of proteasome substrates, induces an unfolded protein response, and ultimately leads to tumor cell death^[68]^. More recently, Eupalinolide B (EB), a natural sesquiterpene lactone, has been identified as another covalent PSMD14 inhibitor. EB covalently binds to PSMD14 at His183 via its reactive α-methylene-γ-lactone moiety, thereby inhibiting its deubiquitinase activity. In acute promyelocytic leukemia cell lines (HL-60), EB promotes AKT1 and CDK4 degradation, leading to G2/M cell cycle arrest and apoptosis. Currently in the early discovery stage, EB represents a novel covalent PSMD14 inhibitor and a promising lead compound for further development^[69]^.

Collectively, these inhibitor studies underscore that the therapeutic efficacy of PSMD14 blockade is fundamentally dictated by its context-dependent substrate networks. Table 2 provides a comprehensive catalog of the characterized PSMD14 substrates, their deubiquitination types, downstream biological effects, and associated drug resistance profiles across different malignancies, offering a molecular rationale for the inhibitor strategies discussed above.

**Table 2:** PSMD14 substrates, downstream effects, and therapeutic resistance across cancer types

**Cancer type**	**Substrate**	**Deubiquitination type**	**Downstream effect**	**Biological process**	**Drug resistance**	**PSMD14 inhibitor**	**Ref.**
Breast cancer (ER+)	ERα	K48-linked	ERα ↑ → ERα target genes (*GREB1*, *TFF1*, *IL20*) ↑	Cell cycle and proliferation	Tamoxifen resistance	THL	[18]
Breast cancer (TNBC)	E2F1	-	E2F1 ↑ → CENPF ↑ → ACSL4 ↓ → ferroptosis suppression ↑	Programmed cell death (ferroptosis); Invasion and metastasis	-	-	[25]
Breast cancer (TNBC)	SF3B4	K63-linked	SF3B4 ↑ → FADS1 ↑ → AA ↑ → Akt/mTOR activation and ferroptosis sensitivity ↑	Programmed cell death (ferroptosis); Metabolic reprogramming; Invasion and metastasis	-	OPA (tool compound)	[26]
Breast cancer	FOXM1	K63-linked	FOXM1 ↑; PI3K/AKT/mTOR activation	Cell cycle and proliferation; Invasion and metastasis	Cisplatin resistance	OPA (tool compound)	[32]
Breast cancer	PD-L1	K48-linked	PD-L1 ↑ → immune evasion ↑	Immune microenvironment	-	-	[41]
Glioblastoma	β-catenin	K48-linked	β-catenin ↑ → Wnt/β-catenin target genes (Axin2, cyclin D1, c-Myc) ↑ → G1/S transition ↑	Cell cycle and proliferation	-	-	[19]
Glioblastoma	BCKDK	K48-linked	BCKDK ↑ → BCAA uptake ↑ → immune evasion ↑	Immune microenvironment; Metabolic reprogramming	-	OPA	[42]
Glioblastoma	IMPDH2	K48-linked	IMPDH2 ↑ → *de novo* purine synthesis ↑ and mitochondrial function ↑ → proliferation and chemoresistance ↑	Cell cycle and proliferation; Mitochondrial function	Temozolomide resistance	THL	[45]
Anaplastic thyroid carcinoma	E2F1	-	E2F1 ↑ → ERK and AKT pathway activation ↑	Cell cycle and proliferation; Invasion and metastasis	-	THL	[20]
Hepatocellular carcinoma	CARM1	K48-linked; K63-linked	CARM1 ↑ → FERMT1 ↑	Cell cycle and proliferation; Invasion and metastasis	-	Capzimin (tool compound)	[21]
Intrahepatic cholangiocarcinoma	ENO1	K63-linked	ENO1 ↑ → ferroptosis suppression ↑	Programmed cell death (ferroptosis); Metabolic reprogramming	-	Capzimin	[27]
Melanoma	SP1	K48-linked	SP1 ↑ → GYS1 ↑	Cell cycle and proliferation; Invasion and metastasis	-	-	[22]
Clear cell renal cell carcinoma	MICALL2	-	MICALL2 ↑	Cell cycle and proliferation; Invasion and metastasis	-	OPA (tool compound)	[23]
Bladder cancer	GPX4	-	GPX4 ↑ → ferroptosis suppression ↑	Programmed cell death (ferroptosis); Cell cycle and proliferation	-	-	[24]
Bladder cancer	Drp1	-	Drp1 ↑ → mitochondrial fission ↑ → proliferation ↑	Mitochondrial function; Cell cycle and proliferation	-	-	[35]
ESCC	SNAIL	-	SNAIL ↑ → E-cadherin ↓ / N-cadherin ↑ / Vimentin ↑ → EMT ↑ → metastasis ↑	Invasion and metastasis	-	-	[29]
ESCC	SNAIL	-	SNAIL ↑ → E-cadherin ↓ / N-cadherin ↑ / Vimentin ↑ → EMT ↑ → metastasis ↑	Invasion and metastasis	Cisplatin resistance	THL	[61]
Lung adenocarcinoma	SMAD3	K48-linked; K63-linked	SMAD3 ↑ → TGF-β ↑ → EMT ↑ → metastasis ↑	Invasion and metastasis	-	THL (tool compound)	[30]
Lung adenocarcinoma	HMMR	K63-linked	HMMR ↑ → TGF-β/Smad and PI3K/AKT/mTOR dual activation → proliferation/metastasis ↑	Invasion and metastasis; Cell cycle and proliferation	-	Capzimin	[65]
Ovarian cancer	PKM2	K63-linked	PKM2 oligomeric state alteration → aerobic glycolysis ↑ and oncogenic gene transcription ↑	Metabolic reprogramming; Invasion and metastasis	-	OPA	[31]
Ovarian cancer	LRPPRC	-	LRPPRC ↑ → autophagy ↓	Mitochondrial function	-	-	[33]
Pancreatic cancer	MYC	-	MYC ↑ → HK2 ↑ → glycolysis ↑	Metabolic reprogramming	-	Capzimin	[36]
Pancreatic cancer	LDHA	K48-linked; K63-linked	LDHA ↑ → lactate ↑ → H3K18la ↑ → ACLY ↑ → lipid synthesis ↑	Metabolic reprogramming	-	THL	[37]
Gastric adenocarcinoma	PFKFB2	K63-linked	PFKFB2 ↑ → lactate ↑ → H3K27la ↑ → SOX9 ↑	Metabolic reprogramming	-	-	[38]
Head and neck squamous cell carcinoma	E2F1	-	E2F1 ↑ → Akt/SOX2 ↑ → stemness and chemoresistance ↑	Cell cycle and proliferation; Invasion and metastasis	Cisplatin resistance	THL	[43]
Colorectal cancer	ALK2	K48-linked	ALK2 ↑ → BMP6 signaling ↑ → ABC transporters ↑ → stemness and chemoresistance ↑	Cell cycle and proliferation	Oxaliplatin resistance; DAPT resistance	-	[44]
Osteosarcoma	-	-	PI3K/Akt/mTOR activation ↑	Cell cycle and proliferation; Invasion and metastasis	Anlotinib resistance	-	[46]
Multiple myeloma	-	-	Proteasome dysfunction → endoplasmic reticulum stress → apoptosis	Programmed cell death (apoptosis)	Bortezomib resistance	OPA	[47]
Multiple myeloma	H2AK119ub (histone)	-	H2AK119ub removal → facilitates H3K36me2 deposition → RELA activation → PSMD14 upregulation (positive feedback)	Epigenetic regulation	Bortezomib resistance	OPA; Capzimin	[48]
Acute promyelocytic leukemia	AKT1; CDK4	-	AKT1/CDK4 ↑ → G2/M progression and apoptosis evasion	Cell cycle and proliferation; Programmed cell death	-	EB	[69]

ERα: Estrogen receptor α; THL: thiolutin; AA: arachidonic acid; Akt: protein kinase B; mTOR: mechanistic target of rapamycin; OPA: O-phenanthroline; PI3K: phosphoinositide 3-kinase; BCAA: branched-chain amino acid; ESCC: esophageal squamous cell carcinoma; EMT: epithelial-mesenchymal transition; MYC: MYC proto-oncogene; LDHA: lactate dehydrogenase A; ACLY: ATP citrate lyase; ABC: ATP-binding cassette; EB: Eupalinolide B.

## DISCUSSION

As a core member of the JAMM family of deubiquitinases, PSMD14 has emerged as a focal point in cancer biology and preclinical research in recent years. This review systematically summarizes the multifaceted regulatory roles of PSMD14 in cell cycle progression, programmed cell death, invasion and metastasis, mitochondrial function, metabolic reprogramming, the immune microenvironment, and therapeutic resistance, while also evaluating its clinical value as a prognostic biomarker and the current landscape of inhibitor development. Taken together, the evidence indicates that PSMD14-targeted therapy has considerable promise in preclinical studies, although several key challenges remain.

For clinical application, PSMD14-targeted intervention faces four major obstacles: inhibitor selectivity and off-target toxicity, suboptimal pharmacokinetic properties, a lack of prospectively validated biomarkers, and the absence of established optimal combination regimens. Currently available inhibitors, including THL^[60]^, Capzimin^[64]^, and OPA^[47]^, chelate the catalytic zinc ion within the JAMM domain. However, because this zinc-binding site is highly conserved among other JAMM family members, the risk of off-target effects is difficult to eliminate completely. In addition, ETPs, although they inhibit PSMD14 through a covalent mechanism, can also target other JAMM family proteins, including CSN5 and AMSH^[68]^. Moreover, most of these compounds exhibit limited bioavailability; only OPA has recently shown preliminary improvement via a nano-delivery system^[67]^. More critically, while PSMD14 expression levels have been correlated with responsiveness to various chemotherapeutic agents, its utility as a predictive biomarker for guiding clinical treatment decisions remains unvalidated by prospective clinical trials, and clinical evidence for optimal combination strategies is largely lacking.

Beyond these challenges, the PSMD14-driven resistance mechanisms in ESCC^[61]^, head and neck squamous cell carcinoma^[43]^, glioblastoma^[45]^, breast cancer^[18]^, and multiple myeloma^[47,48]^ have been relatively well characterized, and pharmacological inhibitors have demonstrated the ability to reverse these resistance phenotypes. These findings suggest that these cancer types represent promising candidate indications for prioritized exploration of PSMD14-targeted therapy.

Based on current evidence, Capzimin appears to be the most promising inhibitor for further preclinical development, with an IC_50_ of 0.34 μM and validated *in vivo* efficacy in solid tumor models including non-small cell lung cancer^[65]^ and prostate cancer^[66]^. OPA has generated the most robust data for reversing bortezomib resistance in multiple myeloma^[47,48]^, and the recent development of a nano-delivery platform has opened new avenues for its therapeutic application^[67]^. THL has demonstrated mechanistic validity and combination potential across multiple solid tumor types^[18,20,43,45,61]^. However, as a natural product, its poor selectivity and moderate potency represent inherent limitations.

In terms of drug discovery strategy, dual-target inhibitor design warrants prioritization. Compound 8b, a PSMD14/HDAC dual inhibitor developed from the THL scaffold, has exhibited superior antitumor activity compared with single-target agents in ESCC^[62]^, suggesting that simultaneous targeting of PSMD14 and epigenetic regulators may offer unique advantages in aggressive solid tumors. Furthermore, based on the role of PSMD14 in stabilizing PD-L1 through deubiquitination, a recent study demonstrated that genetic knockdown of PSMD14 synergizes with anti-PD-1 therapy to exert antitumor effects in breast cancer models^[41]^. Given that pharmacological inhibition can phenocopy the effects of genetic knockdown on PD-L1 stability, we propose the working hypothesis that PSMD14 inhibitors may similarly synergize with anti-PD-1/PD-L1 immune checkpoint blockade, particularly in tumors with high PSMD14 expression levels. This hypothesis warrants systematic evaluation in immunocompetent preclinical models, with priority given to tumors characterized by high PSMD14 expression and pre-existing T cell infiltration. Concurrently, the future clinical application of PSMD14-targeted therapies, if successful, would likely require biomarker-driven patient stratification strategies. We suggest that tumor PSMD14 expression levels be considered a primary stratification criterion for patient enrollment, with PD-L1 status or specific metabolic dependency features further guiding combination regimen selection. However, based on current evidence, the appropriateness of such exclusion criteria requires prospective validation in future clinical studies.

The pathological relevance of PSMD14 extends beyond oncology, as emerging evidence implicates its involvement in various non-tumor diseases. However, research in these areas remains in its early stages. Until substantial breakthroughs are achieved in the oncological setting, exploration of non-tumor indications remains a complementary, albeit worthwhile, direction for future investigation.

## CONCLUSION

PSMD14, as a JAMM-family deubiquitinase, exerts multifaceted regulatory functions in tumorigenesis, progression, metastasis, drug resistance, and immune evasion by stabilizing diverse substrate proteins and has thus emerged as an attractive preclinical therapeutic target and prognostic biomarker. Although existing inhibitors such as THL, Capzimin, and OPA have shown promise in preclinical models, critical issues including selectivity, pharmacokinetic properties, and biomarker validation remain unresolved. Future research should prioritize the advancement of proof-of-concept clinical trials in cancer types such as ESCC, head and neck squamous cell carcinoma, glioblastoma, breast cancer, and multiple myeloma - in which inhibitor-mediated reversal of resistance has already been documented - and systematically explore combination strategies integrating PSMD14 inhibitors with immune checkpoint blockade. The future clinical applicability of PSMD14-targeted therapy ultimately depends on whether these prioritized directions can be validated through biomarker-guided clinical trials.

## DECLARATIONS

### AI and AI-assisted tools statement

During the preparation of this manuscript, the AI tool DeepSeek (version DeepSeek-V3-0324, released March 24, 2025) was used solely for language editing and formatting assistance. The tool did not influence the study design, data collection, analysis, interpretation, or the scientific content of the work. All authors take full responsibility for the accuracy, integrity, and final content of the manuscript.

### Copyright

© The Author(s) 2026.
